# Directional versus ring-mode deep brain stimulation for Parkinson’s disease: protocol of a multi-centre double-blind randomised crossover trial

**DOI:** 10.1186/s12883-023-03387-0

**Published:** 2023-10-18

**Authors:** Timo R. ten Brinke, Hannah Jergas, Vibuthi Sisodia, Michael T. Barbe, Vincent J. J. Odekerken, Dagmar Verbaan, Joke M. Dijk, Maarten Bot, Martijn Beudel, Pepijn van den Munckhof, P. Rick Schuurman, Rob M. A. de Bie

**Affiliations:** 1 Amsterdam UMC, University of Amsterdam, Neurology, Meibergdreef 9, Amsterdam, Netherlands; 2 Amsterdam Neuroscience, Neurodegeneration, Amsterdam, Netherlands; 3 Department of Neurology, University of Cologne, Faculty of Medicine and University Hospital Cologne, Cologne, Germany

**Keywords:** Parkinson’s disease, Deep brain stimulation, Directional deep brain stimulation, Steering subthalamic nucleus

## Abstract

**Background:**

The effectiveness of Deep Brain Stimulation (DBS) therapy for Parkinson’s disease can be limited by side-effects caused by electrical current spillover into structures adjacent to the target area. The objective of the STEEred versus RING-mode DBS for Parkinson’s disease (STEERING) study is to investigate if directional DBS for Parkinson’s disease results in a better clinical outcome when compared to ring-mode DBS.

**Methods:**

The STEERING study is a prospective multi-centre double-blind randomised crossover trial. Inclusion criteria are Parkinson’s disease, subthalamic nucleus DBS in a ‘classic’ ring-mode setting for a minimum of six months, and optimal ring-mode settings have been established. Participants are categorised into one of two subgroups according to their clinical response to the ring-mode settings as ‘responders’ (*i.e.*, patient with a satisfactory effect of ring-mode DBS) or ‘non-responder’ (*i.e.*, patient with a non-satisfactory effect of ring-mode DBS). A total of 64 responders and 38 non-responders will be included (total 102 patients). After an optimisation period in which an optimal directional setting is found, participants are randomised to first receive ring-mode DBS for 56 days (range 28–66) followed by directional DBS for 56 days (28–66) or vice-versa. The primary outcome is the difference between ring-mode DBS and directional DBS settings on the Movement Disorders Society Unified Parkinson’s Disease Rating Scale – Motor Evaluation (MDS-UPDRS-ME) in the off-medication state. Secondary outcome measures consist of MDS-UPDRS-ME in the on-medication state, MDS-UPDRS Activities of Daily Living, MDS-UPDRS Motor Complications–Dyskinesia, disease related quality of life measured with the Parkinson’s Disease Questionnaire 39, stimulation-induced side-effects, antiparkinsonian medication use, and DBS-parameters. Participants’ therapy preference is measured at the end of the study. Outcomes will be analysed for both responder and non-responder groups, as well as for both groups pooled together.

**Discussion:**

The STEERING trial will provide insights into whether or not directional DBS should be standardly used in all Parkinson’s disease DBS patients or if directional DBS should only be used in a case-based approach.

**Trial registration:**

This trial was registered on the Netherlands Trial Register, as trial NL6508 (NTR6696) on June 23, 2017.

## Background

Bilateral deep brain stimulation (DBS) of the subthalamic nucleus (STN) is an established treatment for Parkinson’s disease [1]. The effectiveness of DBS can be limited by adverse-effects such as dysarthria, tonic muscular contractions, and paraesthesia [2]. These adverse-effects can be caused by electrical current spillover into adjacent structures near the motor part of the STN [2, 3]. In theory, the ability to steer the current away from areas which are linked to adverse-effects could help to reduce adverse-effects [4, 5]. By directing the current to the area corresponding to the greatest clinical effect and by avoiding areas which potentially cause adverse-effects, it may also be possible to use greater current amplitudes, potentially improving the efficacy of DBS. Experimental data [3, 6] and studies investigating therapeutic window width [7, 8] indeed suggest that DBS with the availability of directional (*i.e.*, steering) capabilities could have a favourable effect on the performance of DBS. The increased possibilities of directional DBS are accompanied by an increase in programming complexity and likely causes patient follow-up to be more time consuming [9, 10]. Therefore, studies are needed that assess whether the potential clinical improvement of this newer and more complex technology justifies a broad application [9]. In the context of evaluating the potential clinical benefit of directional STN DBS over ring-mode DBS in Parkinson’s disease, we considered three questions especially relevant. Firstly, does directional STN DBS lead to a greater symptom reduction when compared to ring-mode STN DBS in patients who have an unsatisfactory effect of ring-mode setting? Secondly, does directional STN DBS lead to even further symptom reduction when compared to ring-mode STN DBS in patients who already have a satisfactory effect of ring-mode setting? Thirdly, does the entire STN DBS patient population (*i.e.,* the two aforementioned populations pooled together) experience a greater symptom reduction with directional STN DBS when compared with ring-mode STN DBS? This last question is also clinically relevant, as it may lead to standardly implementing directional instead of ring-mode STN DBS from the start of therapy. We initiated a clinical trial to answer these three questions.

## Methods

### Study design

**Fig. 1:**
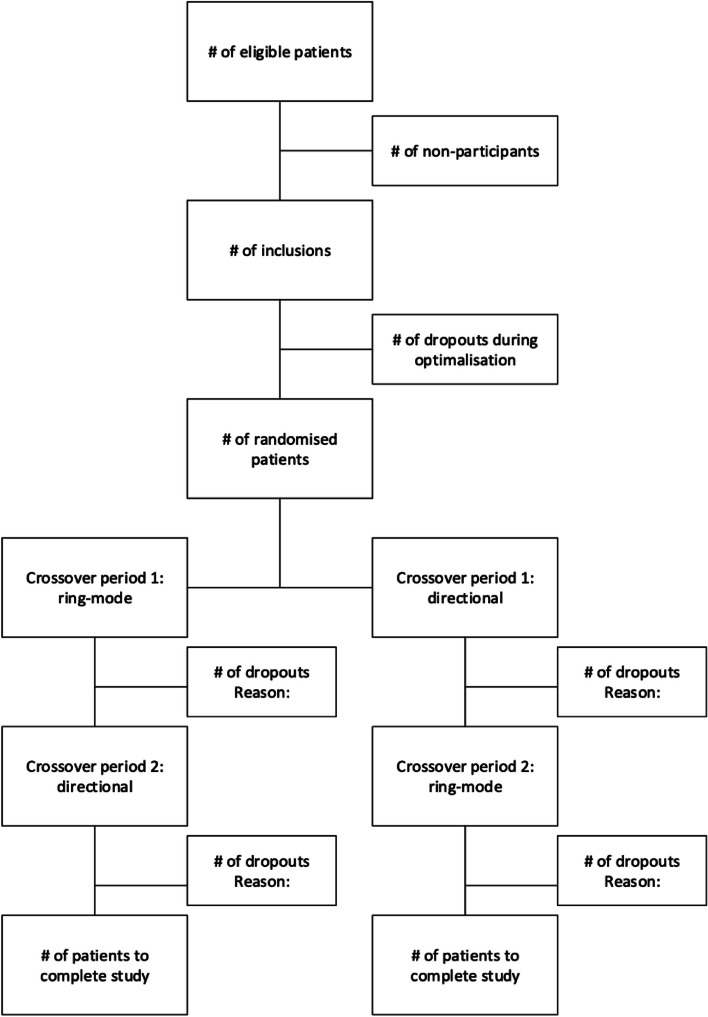
Flowchart of the STEERING trial

We use a multi-centre double-blind randomised crossover trial to assess whether directional STN DBS is more efficacious than ring-mode STN DBS for the treatment of Parkinson’s disease motor symptoms (see Fig. 1). We defined directional DBS as the form of stimulation in which at least one of the two electrodes is programmed to emit electrical current in a specific direction, leading to a non-circular shape of current distribution in the horizontal plane. In effect, any other setting than the even distribution of current between the three contacts on one level (*i.e.*, each of the three contact emits one third of the total current) is considered directional. Ring-mode DBS is the form of stimulation in which the electrical current is emitted omnidirectionally in a horizontal plane. Participants have Boston Scientific® or Medtronic Sensight® directional electrodes implanted for DBS treatment. These electrodes have two contacts from which only omnidirectional current can be emitted (the most ventral and most dorsal contact) and six contacts distributed over two levels which are capable of emitting omnidirectional current (*i.e.*, three contacts are used simultaneously and equally) or emit current in a specific direction (all other configurations).

### Participants

The inclusion criteria are (a) patients with bilaterally implanted Boston Scientific® or Medtronic Sensight® directional electrodes in the STN for the treatment of Parkinson’s disease; (b) at least six months follow-up after implantation of the DBS system; (c) optimal ring-mode DBS settings have been achieved as judged by the physician or nurse specialist and it is anticipated that further adjustment of ring-mode settings will unlikely lead to further motor improvement or would lead to stimulation-induced adverse-effects; and (d) age 18 years and older. Exclusion criteria are (a) legally incompetent; (b) active psychosis; (c) no written informed consent; and (d) no relevant motor symptom suppressing effect in ring-mode DBS on one of the steerable levels.

Patients were recruited from the DBS-clinics of Amsterdam University Medical Centers and the University Hospital of Cologne. Although not formally incorporated in the in- and exclusion criteria, participants had not been subjected to directional DBS before recruitment because this was not applied for Parkinson’s disease in Amsterdam UMC and not in the University Hospital of Cologne other than the previously published cases [11, 12].

### Study procedures and randomisation

If a patient meets the eligibility criteria, the treating neurologist or nurse specialist briefly introduces the study and provides the patient with written information about the study. If the patient is interested in participating, an appointment is made for an extra eligibility check and written informed consent. Each DBS patient is treated by a team consisting of a nurse specialist and a neurologist. Patients were classified as a ‘responder’ (*i.e.*, patient with a satisfactory effect of ring-mode DBS) or a ‘non-responder’ (i.e., patient with a non-satisfactory effect of ring-mode DBS) at the discretion of the treating physician or nurse specialist. The terms satisfactory and unsatisfactory were not further specified.

During the first study visit, the patient is seen in ‘off-medication’ state, defined as a withdrawal from dopaminergic medication for a minimum of 12 h. During the visit, DBS is turned off bilaterally (‘off-stimulation’). In the responder group, patients choose on which side they want the directional stimulation to be performed. Because parkinsonian symptoms are mostly asymmetrical, patients oftentimes have a preference for a specific side. For the non-responders, the researcher together with the patient identifies which electrode is associated with the most bothersome symptoms, based on remaining motor symptoms and/or stimulation-induced adverse-effects. Then, a directional monopolar review is performed on this electrode in which all eight individual contacts of the electrode are tested for rigidity control and adverse-effects thresholds. Depending on the findings and the participant’s preferences, a similar process can (but does not necessarily have to) be repeated for the other electrode at a later study visit prior to randomisation. As a result, the directional DBS setting of each participant can consist of one electrode with directional current configuration and one electrode with ring-mode configuration, or of two electrodes with directional current configuration. For the final directional setting before randomisation, at least one of the electrodes must be programmed in a directional current configuration. It is possible that an electrode which was used for monopolar directional review is still used in a ring-mode configuration in the final directional setting.

During the directional monopolar review, each of the eight contacts on the electrode are tested by slowly increasing the amplitude whilst measuring rigidity on the contralateral wrist. If rigidity cannot be adequately measured, other symptoms such as tremor or bradykinesia are used to determine the efficacy threshold. Once this threshold has been determined, the current is slowly increased further until an amplitude is reached which causes an intolerable stimulation-induced adverse-effect (this excludes symptoms such as transient paraesthesia, which tend to abate in the course of minutes without alteration of stimulation settings). The interval between the efficacy threshold and the adverse-effect threshold is considered the therapeutic window for the tested contact. Upon completion of this process for all eight contacts of the electrode, one to three new ‘directional programs’ are programmed into the device, based on therapeutic window width and efficacy threshold data from the directional monopolar review, complemented by clinical insight from testing multiple different stimulation configurations. In these new programs, margins for adjusting stimulation amplitude are set-up for later adjustments. Additionally, the ring-mode program that the patient had before enrolment in the study is kept as one of the programs on the device, which can be used as an ‘escape option’ by the patient if the newly programmed configurations have a worse clinical effect than the ring-mode program. Other DBS-parameters such as frequency and pulse width are not changed during the course of the trial.

After this initial visit, an optimisation period is implemented. In order to reflect the normal clinical practice in DBS programming for Parkinson’s disease, no formal duration or visit schedule is established for this period. Instead, visits and telephone consultations are planned as needed. Changes in stimulation parameters are recorded. The end of the optimisation period is reached when the researcher and the patient agree that the new – directional – stimulation program is as good or better than the ring-mode program, and further programming efforts are not likely to lead to a substantial improvement of Parkinson’s disease symptoms. When this program is considered to be stable (as a rule of thumb a period of at least two weeks without the need for adjustments is considered stable), the patient can be randomised.

At the randomization visit, the researcher records their prediction of whether or not the directional program will lead to a greater improvement of Parkinsonian symptoms when compared to the ring-mode. Patients are randomised into a crossover study design using ALEA randomisation software (www.aleaclinical.eu). Allocation is set to a 1:1 ratio with block sizes of two and four. Patients are stratified by their responder/non-responder status. Each participating patient receives both directional and ring-mode DBS treatment for a period of two months (56 days, with an accepted range of 28 to 66). Patients are randomised to receive directional stimulation as the first intervention and ring-mode stimulation as the second (directional-first group) or to receive the two interventions in reversed order (ring-mode-first group). The physician co-investigators and nurse specialists performing the programming are not blinded to patient allocation. Participants and trial nurses doing the assessments are blinded to the DBS settings (*i.e.*, ring-mode or directional DBS). The statistician analysing the results of the trial will be blinded as well.

At the end of the first intervention visit (*i.e.*, before cross-over) and at the end of the second intervention visit (*i.e.*, at the end of follow-up) the primary and secondary outcome measurements and questionnaires are performed. At the end of the second intervention, whilst still in blinded condition, the patient chooses which of the two programs they want to keep: the first or second intervention. After the participant finished all assessments, they may be unblinded and return to regular DBS follow-up.

Approval of the medical ethical committee of both participating centres was obtained. The STEERING-study conforms to the principles of the Declaration of Helsinki (version 2008). Study monitoring and data management are performed in accordance with the International Conference on Harmonisation—Good Clinical Practice guidelines. It is an investigator-initiated study. Funding organizations did not have an influence on the design, execution, data analysis, or participation in article drafting.

### Outcome measures

The outcome measures that will be assessed in the STEERING trial are summarized in Table 1. The primary outcome is the difference on the Movement Disorders Society Unified Parkinson’s Disease Rating Scale – Motor Evaluation (MDS-UPDRS-ME) [13] score in off-medication state with stimulation turned on between ring-mode DBS and directional DBS setting.

**Table 1:** Outcome measures

**Outcome measure**	**Domain**	**Best score**	**Worst score**
MDS-UPDRS-ME	Motor symptoms	0	132
MDS-UPDRS-ADL	Motor aspects in Activities of daily living	0	52
MDS-UPDRS-MC-A	Dyskinesias	0	8
PDQ-39	Quality of life	0	100
LED	Medication use	N/A	N/A
Stimulation settings	Stimulation	N/A	N/A
Likert scale about quality of walking	Motor symptoms	1	5
Likert scale about speech	Motor symptoms and adverse-effects	1	5
Adverse-effects	Adverse-effects	N/A	N/A
Therapy choice at end of trial	N/A	N/A	N/A

*Led* levodopa equivalent dose, *MDS-UPDRS-ADL* Movement Disorders Society Unified Parkinson’s Disease Rating Scale Activities of Daily Living, *MDS-UPDRS-MC-A* Movement Disorders Society Unified Parkinson’s Disease Rating Scale Motor Complications–Dyskinesia, *MDS-UPDRS-ME* Movement Disorders Society Unified Parkinson’s Disease Rating Scale – Motor Evaluation, *N/A* Not applicable, *PDQ-39* Parkinson’s Disease Questionnaire 39

Secondary outcome measures consist of MDS-UPDRS-ME in on-medication state with stimulation turned on, MDS-UPDRS Activities of Daily Living (MDS-UPDRS-ADL) [13], MDS-UPDRS Motor Complications–Dyskinesia (MDS-UPDRS-MC-A) [13], disease related quality of life measured with the Parkinson’s Disease Questionnaire 39 (PDQ39) [14], stimulation-induced adverse-effects, antiparkinsonian medication use expressed in Levodopa Equivalent Dose (LED) [15], two 5-point Likert scales concerning quality of walking and of speech and DBS-parameters. As stated before, patients’ therapy preference is measured at the end of the study.

### Statistics

The sample size calculation was carried out separately for the responder group and the non-responder group. The minimum clinically important differences between ring-mode and directional DBS for the responder and non-responder groups, respectively, were three points and five points on the MDS-UPDRS-ME [16, 17]. For the responder group, we calculated a required sample size of 58 patients (29 patients in each sequence group) to detect an intra-individual difference of three points on the MDS-UPDRS-ME in off-medication state with an assumed intra-individual standard deviation (SD) of 10, 80% power and at 5% significance level. For the non-responder group, we calculated a required sample size of 34 patients (17 patients in each sequence group) to detect an intra-individual difference of five points on the MDS-UPDRS-ME in the off-medication state with an assumed intra-individual standard deviation (SD) of 10, 80% power and at 5% significance level. Considering a drop-out rate of 10% in each group, 64 responders and 38 non-responders will be included. The total sample size will be 102 patients.

Statistical analysis will be based on the intention-to-treat principle. In all analyses, statistical uncertainties will be expressed in 95% confidence intervals (CI). The primary outcome is the difference between ring-mode and directional DBS settings on the off-medication state MDS-UPDRS-ME. This analysis will be performed for responder and non-responder groups separately and for the total study population (*i.e.*, responders and non-responders pooled). Paired t-tests will be used to assess these differences, or, if not normally distributed, a Wilcoxon signed-rank test. Differences will be considered significant at *p* < 0.05 without correcting for multiple comparisons.

It is unlikely that the cross-over will have a sequence effect since the primary outcome is an objective outcome measure and will not be influenced by the patient's perspective. However, the possibility of a sequence effect cannot be ruled out. A possible sequence effect will be evaluated descriptively, as a statistical analysis cannot verify this effect [18].

We will use mixed model analysis and per-protocol analyses to evaluate the robustness of the results and to address the issue of missing data. Patients will be excluded from the per-protocol analyses if they receive the incorrect form of stimulation after randomisation (*e.g.*, patients who change the by randomisation allocated stimulation parameters) or if they do not have the outcome assessments at the pre-specified time window (56 days, with an accepted range of 28 to 66) from randomisation and cross-over visit, respectively.

Other secondary outcomes consisting of on-medication state administered MDS-UPDRS-ME, MDS-UPDRS-ADL, MDS-UPDRS-MC-A, PDQ39, and LED will be presented as point estimates with unadjusted 95% confidence intervals without *p*-values. Patient preference, two 5-point Likert scales concerning quality of walking and of speech, and stimulation-induced adverse-effects will be compared between the ring-mode and directional DBS settings with appropriate non-parametric statistics.

Baseline values and the stimulation parameters of the ring-mode and directional-mode will consist of both categorical (*e.g.*, sex, occurrence of stimulation induced adverse-effects) and continuous variables (*e.g.*, age, disease duration, LED). These data will be presented quantitatively in tables.

## Discussion

Due to the broad distribution of recent advances in DBS technology, the implementation of directional DBS is feasible in standard care for Parkinson’s disease patients. This may lead to an increase in programming time and effort, possibly constituting an added burden for patients and clinicians alike [9]. For clinical practice, it would be of added value to know whether directional DBS leads to a clinical improvement in patients who already experience a good effect from ring-mode DBS, and in patients who experience a sub-optimal effect from ring-mode DBS. In order to investigate whether the use of directional STN DBS has a more beneficial effect on the motor symptoms of Parkinson’s disease when compared to ring-mode DBS, we use a randomised, double-blind crossover trial design. Some of the choices made in the study protocol warrant discussion.

First, we discuss performing three separate analyses for responders, non-responders and these two study populations pooled together. It is possible that there is a difference in added value of directional STN DBS when compared to ring-mode STN DBS for responders but not for non-responders, or vice-versa. Another possibility is that both responders and non-responders have added benefit from directional STN DBS, but to a different degree. Pooling responders and non-responders allows for a representative sample of the entire patient population of patients with Parkinson’s disease who received DBS treatment. If a significant difference is found between directional and ring-mode settings in the favour of directional STN DBS, this would mean we could implement directional STN DBS as the new treatment standard. Additionally, the pooled analysis will have greater power due to the higher number of subjects.

A second relevant issue is that it was not required for randomisation to have directional setting bilaterally. This approach was chosen because the most often cited reason to employ directionality is the occurrence of stimulation-induced adverse-effects [9]. These adverse-effects are not necessarily present at both sides within therapeutic current amplitudes. Another reason to opt for this approach is that a comprehensive directional monopolar review is a time-consuming effort, which can be burdensome for the patient. Hence, we anticipated that this approach warrants the best external validity.

Third, a randomised double-blind crossover trial design was chosen to answer the research question: each patient will receive both directional and ring-mode DBS treatment for a period of two months (56 days) with an accepted range of 28 to 66 days. This range was chosen, as some patients found it difficult to continue with a DBS stimulation setting (*e.g.*, ring-mode DBS) for two months if the other setting (*e.g.*, directional DBS) felt significantly better. The aim of the current study is that all patients receive a DBS setting for two months; however, if they are unable to endure the DBS stimulation setting or if there are logistical issues, then these patients will be assessed earlier. In any crossover trial, the possibility of carry-over effect should be considered. In STN DBS for Parkinson’s disease, relatively short washout periods have been reported for motor symptoms such as bradykinesia. The UPDRS motor score of STN DBS Parkinson’s disease patients was shown to worsen 90% after two hours in medication and stimulation-off condition, with axial signs worsening after three to four hours [19]. In addition to this ‘fast washout’, a slower decline of the residual effect in off-stimulation condition may occur [20]. Little is known about possible longer lingering effects. We do not implement a washout period in off-stimulation condition in this study, as we expect most of the clinical effects of the previous setting to wane within the first hours of stimulation switch. We expect that most, if not all carry-over effects will have disappeared after the minimum follow-up period of four weeks. We expect that the used settings will have had a representative effect on the participants’ symptoms, adverse-effects, and quality of life in each of the treatment periods in the crossover setting.

In the current study, only the used contact configuration, polarity and current amplitude can be adjusted. Pulse width and pulse frequency are not adjusted during the trial. In clinical practice, situations may occur when all these variables are changed in the optimisation process. However, adjusting these variables would hinder judgement of the net effect of directional DBS. Directional stimulation settings were found through the method of directional monopolar review and adjusted according to clinical insights. No imaging techniques such as prediction of volume of tissue activated (VTA) or diffusor tensor imaging (DTI) were used to assist on determining the stimulation settings.

The possible outcomes of the STEERING-trial are likely to have clinical consequences. If a beneficial effect of directional DBS is demonstrated in the responder subgroup, this means that good responders can do even better with directional DBS. If non-responders perform better with directional DBS, this might mean that’non-response’ can be caused by suboptimal electrode placement or limiting adverse-effects. If a beneficial effect of directional DBS is demonstrated in the responder subgroup, an argument can be made for performing a directional monopolar review in all patients in the weeks/months following surgery, to then implement directional stimulation standardly. If no beneficial effect of directional DBS is demonstrated, the current method of using ring-mode DBS on devices capable of directional stimulation can be continued. Directionality will still be an important tool for troubleshooting in individual patients, as its usefulness in these conditions has been previously described [11].

## Data Availability

Depending on the type of data and associated privacy regulations, data from the STEERING project will be made publicly available or will become available via the corresponding author, upon reasonable request.

## Abbreviations

CI: Confidence intervals
DBS: Deep brain stimulation
DTI: Diffusor tensor imaging
LED: Levodopa equivalent dose
MDS-UPDRS: Movement Disorders Society Unified Parkinson's Disease Rating Scale
MDS-UPDRS-ADL: Movement Disorders Society Unified Parkinsons' Disease Rating Activities of Daily Living Scale
MDS-UPDRS-MC-A: Movement Disorders Society Unified Parkinson's Disease Rating Scale Motor Complications – Dyskinesias
MDS-UPDRS-ME: Movement Disorders Society Unified Parkinson's Disease Rating Motor Examination Scale
PDQ39: Parkinson’s Disease Questionnaire 39
STN: Subthalamic nucleus
VTA: Volume of tissue activated
